# Reproducible Polybutylene Succinate (PBS)-Degrading Artificial Consortia by Introducing the Least Type of PBS-Degrading Strains

**DOI:** 10.3390/polym16050651

**Published:** 2024-02-28

**Authors:** Nara Shin, Su Hyun Kim, Jinok Oh, Suwon Kim, Yeda Lee, Yuni Shin, Suhye Choi, Shashi Kant Bhatia, Yun-Gon Kim, Yung-Hun Yang

**Affiliations:** 1 Department of Biological Engineering, College of Engineering, Konkuk University, Seoul 05029, Republic of Korea; dksk71@naver.com (N.S.); gsm06136@naver.com (S.H.K.); xmfvm@naver.com (J.O.); rlatn990@naver.com (S.K.); karecurry@konkuk.ac.kr (Y.L.); sdbsdl0526@naver.com (Y.S.); suhye0823@konkuk.ac.kr (S.C.); shashikonkukuni@konkuk.ac.kr (S.K.B.); 2 Institute for Ubiquitous Information Technology and Application, Konkuk University, Seoul 05029, Republic of Korea; 3 Department of Chemical Engineering, Soongsil University, Seoul 06978, Republic of Korea; ygkim@ssu.ac.kr

**Keywords:** bioplastic, polybutylene succinate, degradation, bacterial consortia, artificial consortia

## Abstract

Polybutylene succinate (PBS) stands out as a promising biodegradable polymer, drawing attention for its potential as an eco-friendly alternative to traditional plastics due to its biodegradability and reduced environmental impact. In this study, we aimed to enhance PBS degradation by examining artificial consortia composed of bacterial strains. Specifically, *Terribacillus* sp. JY49, *Bacillus* sp. JY35, and *Bacillus* sp. NR4 were assessed for their capabilities and synergistic effects in PBS degradation. When only two types of strains, *Bacillus* sp. JY35 and *Bacillus* sp. NR4, were co-cultured as a consortium, a notable increase in degradation activity toward PBS was observed compared to their activities alone. The consortium of *Bacillus* sp. JY35 and *Bacillus* sp. NR4 demonstrated a remarkable degradation yield of 76.5% in PBS after 10 days. The degradation of PBS by the consortium was validated and our findings underscore the potential for enhancing PBS degradation and the possibility of fast degradation by forming artificial consortia, leveraging the synergy between strains with limited PBS degradation activity. Furthermore, this study demonstrated that utilizing only two types of strains in the consortium facilitates easy control and provides reproducible results. This approach mitigates the risk of losing activity and reproducibility issues often associated with natural consortia.

## 1. Introduction

Plastic waste pollution has emerged as a critical global environmental issue primarily due to the high chemical resistance and slow degradation of plastics [1,2]. According to the World Bank, annual global waste generation is projected to reach 3.40 billion tons by 2050, a significant increase from the current 2.01 billion tons [3]. Despite the convenience and indispensability of plastics in daily life and industry, inadequate disposal methods and the enduring physical properties of plastic products have led to substantial environmental challenges, including soil contamination and the disruption of marine ecosystems [4]. Furthermore, plastic waste persists for prolonged periods in the environment, with a degradation period spanning thousands of years [5,6]. Nevertheless, recent studies have indicated that the microbial degradation of plastic holds promise as a potential solution to this issue [7]. The accumulation of plastic waste has emerged as a prominent global environmental concern, prompting growing interest in sustainable alternatives, such as biodegradable plastics [8].

Bioplastics have gained popularity as viable alternatives to conventional plastics, primarily due to their potential to mitigate the adverse environmental impacts associated with petroleum-based plastic waste [9]. Biodegradable plastics are specifically engineered to decompose into natural compounds such as water, carbon dioxide, and organic matter through biological processes [10]. Additionally, they may provide enhanced support for microorganisms and offer more microbial attachment sites than traditional plastics [11]. However, certain bioplastics may still significantly contribute to global warming, pollution, and substantial land-use changes [12]. Despite this, bioplastics offer numerous advantages in reducing pollution and exhibit superior mechanical properties compared to traditional plastics [13].

Polybutylene succinate (PBS) has garnered significant attention as a bioplastic material in recent years. PBS possesses a melting point of 115 °C, which is lower than that of polylactic acid (PLA) [14]. This characteristic presents potential savings in industrial processing, as it necessitates less time for melting and allows for easy blending with other materials [15]. Furthermore, PBS is facile to process and handle when combined with other substances. Additionally, PBS exhibits favorable thermal stability and impressive mechanical properties [16]. As a sustainable alternative to petrochemical-derived plastics, PBS offers properties akin to widely utilized low-density polyethylene (LDPE), high-density polyethylene (HDPE), and polypropylene (PP), commonly employed in short-shelf-life products such as packaging and mulch films [17]. Notably, PBS has a lower carbon footprint than traditional petroleum-based plastics and finds applications in various sectors, including packaging and agricultural films [18,19].

Several studies have focused on the degradation of PBS, a material commonly used in everyday applications, due to the action of individual bacterial strains. However, research on PBS degradation by consortia comprising multiple bacterial strains is scarce (Table 1) [20,21,22,23,24,25]. Various species of *Bacillus* and *Terribacillus*, isolated from environmental sources, are known for their ability to degrade different types of polyester plastics.

When organized into consortia, numerous microorganisms demonstrate enhanced efficiency in degrading diverse types of plastic polymers at a faster pace than when employed as pure cultures [26]. Within microbial communities, microorganisms compete for finite nutrients and utilize the metabolic products released by other species to enhance their fitness [27]. Consortia of microbes degrade polymer compounds via co-metabolism or degradation [28]. Metabolic interactions significantly influence the practical implementation of microbial consortia. Substances released by species can either bolster or inhibit the growth of other species, modify their interactions, or potentially affect the overall functioning of an entire community [29]. However, the absence of a rational design for microbial consortia, in contrast, is a hurdle to realizing the potential of microbial consortia [30].

In the present study, consortia were formed by combining *Teribacillus* sp. JY49, a known PBS-degrading strain, with *Bacillus* sp. NR4 and *Bacillus* sp. JY35, recognized for their proficiency in degrading polybutylene adipate-*co*-terephthalate (PBAT) and polycaprolactone (PCL). The most effective consortium for PBS degradation was subsequently identified. Furthermore, the study delved into the advantages of employing a microbial consortium and compared them with those of a single strain. Consequently, two potential species, *Bacillus* sp. NR4 and *Bacillus* sp. JY35, were chosen based on their capability to degrade PBS. Additionally, alterations in the physical properties, chemical composition, and the degradation activities of the consortium on various plastics were studied.

## 2. Materials and Methods

### 2.1. Chemicals

All chemicals used in this study were of analytical grade. Chloroform and dichloromethane (DCM) were procured from Junsei Chemical Co. (Tokyo, Japan). Polycaprolactone (PCL) and polylactic acid (PLA) were purchased from Sigma-Aldrich (St. Louis, MO, USA). Polyhydroxybutyrate (PHB) pellets were obtained from Goodfellow Cambridge Ltd. (Huntingdon, UK). Polybutylene succinate (PBS) pellets were sourced from ANKOR Bioplastics Co., Ltd. (Wonju, Republic of Korea). Polybutylene adipate terephthalate (PBAT) was obtained from SK Ecovance (Seoul, Republic of Korea). Poly-3-hydroxybutyrate-*co*-4-hydroxybutyrate (P(3HB-*co*-4HB)) pellets were procured from CJ (Suwon, Republic of Korea).

### 2.2. Formation of PBS Film and Solid Plate Containing Bioplastics Using Solvent Casting

The process of preparing PBS films involved utilizing the solvent-casting process. Initially, 0.5 g of PBS pellets were dissolved in 250 mL of chloroform and then heated in a water bath set to 60 °C. After fully dissolving the pellets, the resultant solution was poured onto a Petri dish and allowed to stand in a fume hood at room temperature until the solvent totally evaporated.

To prepare a solid plate containing bioplastic emulsion, 0.5 g of each pellet of PBS, PBAT, PCL, PLA, PHA, PHB, and P(3HB-*co*-4HB) was added to 20 mL of DCM and dissolved in a water bath at 60 °C until the pellet was completely dissolved. Thereafter, 50 mL of distilled water (DW) was added to the lysate, and 1 mL of 2% Sarkosyl NL was added to the interface. The mixture was subjected to sonication using a Vibra-Cell VCX500 (Sonics & Materials, Inc., Newtown, CT, USA) with 10 s of pulsing at 30% amplitude for a duration of 10 min. Thereafter, 2% agarose and 1 g/L bioplastic emulsion were added to the marine broth (MB; Difco Laboratories, Detroit, MI, USA) medium, and this mixture was sterilized by autoclaving at 120 °C for 15 min.

### 2.3. Formulating Microbial Consortia for PBS Degradation

To identify a consortium capable of effectively degrading PBS, strains capable of PBS degradation were selected (Table 2). *Terribacillus* sp. JY49 was isolated from marine samples and exhibited degradability to PBS [22]. *Bacillus* sp. JY35 was isolated from wastewater sludge and had high PBAT degradability [31]. *Bacillus* sp. NR4 was isolated from marine environments and was degradable to PCL [32]. Three bacterial strains were selected: *Terribacillus* sp. JY49, exhibiting high activity toward PBS; *Bacillus* sp. JY35, with low degradation activity; and *Bacillus* sp. NR4, exhibiting almost no degradation activity. The effect of *Bacillus* sp. NR4 on the degradation activity toward PBS was confirmed through various combinations of consortia.

### 2.4. Quantification of Degradation Yield through Liquid Culture

In the liquid culture method, PBS films were sectioned into 20 mg fragments and subjected to sterilization using 70% ethanol and UV radiation within a sterile environment. Subsequently, *Terribacillus* sp. JY49, *Bacillus* sp. JY35, and *Bacillus* sp. NR4 cells were inoculated into 40 mL of MB liquid medium in a 100 mL flask, along with the sterile film fragments. The degradation rate over time was assessed at 3, 5, 7, and 10 days of cultivation. In this study, 2% of *Terribacillus* sp. JY49, *Bacillus* sp. JY35, and *Bacillus* sp. NR4 cells were used, and the cultures were agitated on a rotary shaker at 200 rpm. The remaining film fragments were recovered, washed with distilled water, and freeze dried prior to further experiments.

### 2.5. Monitoring of the Clear Zone by Solid Culture

To analyze and optimize the characteristics of single strains and consortia of microorganisms capable of degrading PBS, clear-zone tests were conducted. The bacterial consortium was cultured in an optimal liquid medium for 24 h at 30 °C to determine if it produced a clear zone. Paper discs (Toyo Roshi Kaisha, Tokyo, Japan) were then placed on the plate [32], and 10 μL of the cultured cells were inoculated onto the paper disc and incubated at 30 °C for 14 days. The radius of the clear zones was measured by determining the distance between the paper disc and the edge of the clear zone. All experiments were carried out in duplicate, and quantification of protein concentration in microbial pre-culture supernatant was performed using the Bradford assay.

### 2.6. Quantification of Protein Concentration in Microbial Pre-Culture Supernatant through Bradford Assay

The protein concentration in the supernatant of the microbial pre-culture was assessed using the Bradford assay. A 1:5 dilution of Coomassie Brilliant Blue G-250 (Bio-Rad, Hercules, CA, USA) was prepared in 95% ethanol, and then combined with 0.1 M phosphate buffer (pH 7.0) in a 1:4 ratio to create the final working solution. Bovine serum albumin (BSA) standards ranging from 0 to 1 mg/mL were prepared and mixed with the Bradford reagent in cuvettes. The absorbance was measured at 595 nm using a UV-Vis spectrophotometer (Agilent, Santa Clara, CA, USA) to generate a standard curve. Microbial pre-culture supernatant samples were mixed with the Bradford reagent in cuvettes, and the absorbance was measured at 595 nm after allowing the reaction to proceed for 5 min at room temperature. The protein concentrations of the samples were determined using a standard curve and calculated using a linear regression equation obtained from the BSA standard curve [33].

### 2.7. Analysis of PBS Degradation Yield Using Gas Chromatography–Mass Spectrometry (GC–MS)

After degradation, the degradation yield and residual count analysis of PBS were performed using GC–MS (YOUNG IN ChroMass, Anyang, Republic of Korea) using a fused silica capillary column (30 m × 0.25 mm i.d. × 0.25 μm). GC–MS analysis samples were prepared through fatty acid methyl ester (FAME) derivatization [34]. Methanolysis was performed by adding 1 mL of methanol/sulfuric acid (85:15 *v*/*v*) and 1 mL chloroform to the degraded PBS film and heating at 100 °C for 120 min. The samples were cooled at room temperature, and 1 mL of HPLC-grade water was added to the samples and vortexed to mix well. Subsequently, the organic phase layer of the sample was placed in anhydrous sodium sulfate, and the residue was removed. The samples then underwent a linear temperature gradient for examination, beginning at 50 °C for 1 min and linearly increasing at 15 °C/min until they reached 120 °C, where they stayed for another 2 min. Subsequently, the temperature was raised at 10 °C per min until reaching 300 °C, where it remained for 10 min [35]. The injector port temperature was maintained at 250 °C. Mass spectra were generated using electron impact ionization at 70 eV, and the scan spectra were recorded within the range of 45–450 *m*/*z*. A calibration curve was established to estimate the quantity of residual PBS films.

### 2.8. Derivatization Method

The derivatized PBS degradation products were analyzed using GC–MS (YOUNG IN ChroMass, Anyang, Republic of Korea). Derivatized samples were analyzed using GC–MS equipment [36]. Derivatization was performed using BSTFA containing 1% TMCS from Sigma-Aldrich (St. Louis, MO, USA). The derivatizing agents, as well as the individual and composite standard solutions, were stored at 4 °C. After PBS degradation, the supernatant was dried using a freeze dryer (ilShinBioBase, Dongducheon, Republic of Korea), and then BSTFA was added to react at 60 °C for 1 h and 30 min. The reacted derivatized extract was transferred to a vial and analyzed by GC–MS.

### 2.9. Characterization and Analysis of PBS Films after Degradation

#### 2.9.1. Scanning Electron Microscopy (SEM)

Surface changes in the PBS films were analyzed using scanning electron microscopy (SEM). The films were degraded by the *Bacillus* sp. JY35 and *Bacillus* sp. NR4 consortium for 0, 5, and 10 days, and the residual films were collected daily. They were washed with distilled water to remove the medium components and lyophilized. Subsequently, the PBS films were coated with gold dust at 5 mA for 300 s, and backscattered electron images were obtained using a TM4000Plus SEM instrument (Hitachi, Tokyo, Japan) at a voltage of 5 kV [37].

#### 2.9.2. Fourier Transform Infrared Spectroscopy (FT-IR)

The PBS films exposed to bacterial consortia for 10 days were analyzed using an FTIR spectrometer (PerkinElmer, Spectrum Two, MA, USA). Subsequently, the samples were washed with distilled water and air dried. The plastic films were then cut into 1 cm × 1 cm pieces and thoroughly mixed with 250 mg of potassium bromide (KBr). This mixture was homogenized, placed in a stainless steel vial, frozen using liquid nitrogen, and agitated. An additional 350 mg of KBr was added to the mixture, and the components were carefully pressed to form a KBr disk for further analysis. To eliminate background noise, a blank scan was conducted within the frequency range of 4000 to 500 cm^−1^. Similarly, the samples were scanned within the regions of 400–4000 cm^−1^ at a resolution of 4 cm^−1^. The resulting spectrum was presented as a plot of the percentage transmittance versus the wavenumber and subsequently compared against the respective controls.

#### 2.9.3. Gel Permeation Chromatography (GPC)

The molecular weight changes in the degraded PBS films were analyzed using gel permeation chromatography (GPC) equipment (YOUNG IN ChroMass, Anyang, Republic of Korea). To prepare the samples, the residual PBS films were dissolved in chloroform and heated at 60 °C for 1 h. The resulting solution was then filtered using a syringe filter (0.2 μm pore size; Chromdisc, Daegu, Republic of Korea). The analysis was conducted using an HPLC apparatus, including a loop injector (Rheodyne 7725i), an isocratic pump with dual heads (YL9112), a column oven (YL9131), columns (Shodex, K-805, 8.0 mm I.D. × 300 mm; Shodex, K-804, 8.0 mm I.D. × 300 mm), and a refractive index detector (YL9170). A 60 μL sample was injected for analysis. Chloroform served as the mobile phase, with a flow rate of 1.0 mL/min and a temperature of 35 °C. The data were processed using the YL-Clarity software version 8.3 for a single YL HPLC instrument (YOUNG IN ChroMass). The molecular weight was determined relative to polystyrene standards ranging from 5000 to 2,000,000 g/mol [38,39].

## 3. Results and Discussion

### 3.1. Evaluation of Degrading Ability by Artifitial PBS Degrading Bacterial Consortia

Plastic degradation through naturally occurring consortia offers numerous advantages, yet it is not without its limitations. The degradation of PBS, whether through soil burial or indigenous consortia, is characterized by prolonged degradation periods or suboptimal degradation efficiency. The accurate prediction or control of natural consortia behavior proves challenging due to the intricate interactions among diverse microorganisms [40]. Furthermore, the degradation efficiency of plastics within natural consortia can be significantly influenced by environmental changes, leading to low reproducibility [41]. Previous studies on PBS degradation have reported degradation rates of 60.7% when PBS films underwent controlled composting conditions for 90 days. Following degradation, *Aspergillus vericolor*, *Penicillium*, *Bacillus*, and *Thermopolyspora* were isolated from the compost, which seems to have affected PBS degradation [25]. To enhance the PBS degradation rate within a shorter timeframe and establish reproducible consortia, we investigated PBS degradation by creating artificial consortia that mimic the consortia existing in the nature. The microorganisms employed to construct these artificial consortia were *Bacillus* sp. JY35, *Terriacillus* sp. JY49, and *Bacillus* sp. NR4. *Bacillus* sp. JY35 demonstrated degradation in PBAT. *Terriacillus* sp. JY49 exhibited degradability in PBS. *Bacillus* sp. NR4 showed degradability in both PCL and PBAT.

**Figure 1:**
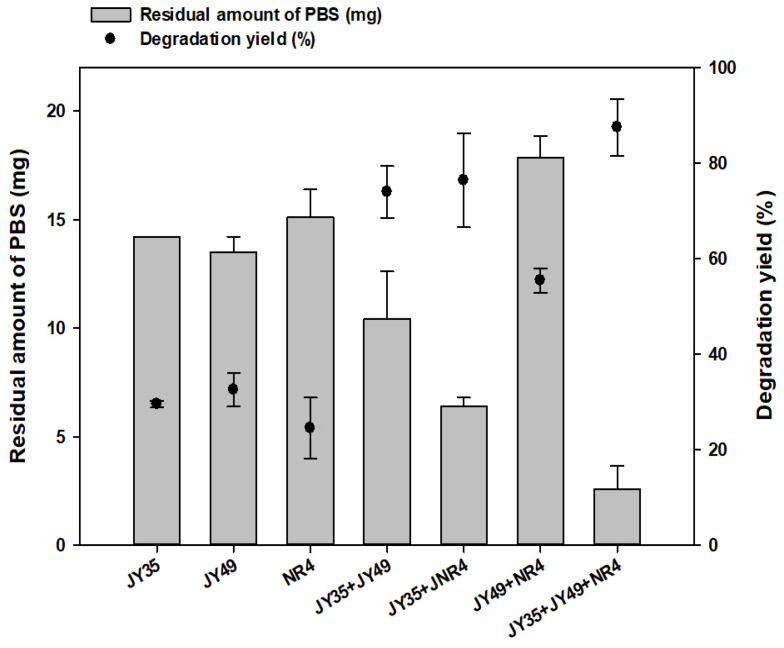
Comparison of PBS degradation ability among the screened isolates. The degradation yield (%) of PBS films when cultured in liquid media at 30 °C for 10 days.

The liquid culture analysis aimed to assess the capacity of bacterial consortia in degrading PBS. The degradation rate of PBS films was determined through gas chromatography–mass spectrometry (GC–MS). Single strains (*Bacillus* sp. JY35, *Terribacillus* sp. JY49, *Bacillus* sp. NR4), along with four consortia, were cultivated in liquid MB, each with 20 mg of PBS films, over a 10-day period at 30 °C. Liquid-based methods facilitated the identification of strains based on their degradability (Figure 1). Comparing GC–MS results revealed that a combination of *Bacillus* sp. JY35, *Terribacillus* sp. JY49, and *Bacillus* sp. NR4 exhibited the highest degradation yield (87.4%). Surprisingly, when a single strain, co-cultivating *Bacillus* sp. JY35 and *Bacillus* sp. NR4, was used, despite the latter not displaying high activity against PBS degradation, it showed the second-highest degradation activity at 76.5%. Among the formulated consortia, the highest PBS degradation yield was observed in the mixture of three strains: *Bacillus* sp. JY35, *Terribacillus* sp. JY49, and *Bacillus* sp. NR4. However, this study primarily conducted an overall PBS degradation experiment using a consortium of *Bacillus* sp. JY35 and *Bacillus* sp. NR4, which exhibited a notably high degradation yield (twice). This choice aimed to efficiently degrade PBS using consortia composed of the fewest strains while ensuring reproducibility.

### 3.2. Comparison of Single Strain and Consortia Degradability on PBS in Solid Media and Measurement of Secreted Protein

A clear zone test was conducted as a simple semi-quantitative method to confirm the decomposition activity of microorganisms on plastic. This method is commonly used to identify organisms capable of degrading a specific polymer but can also yield approximate results by examining the development of transparent areas [42].

**Figure 2:**
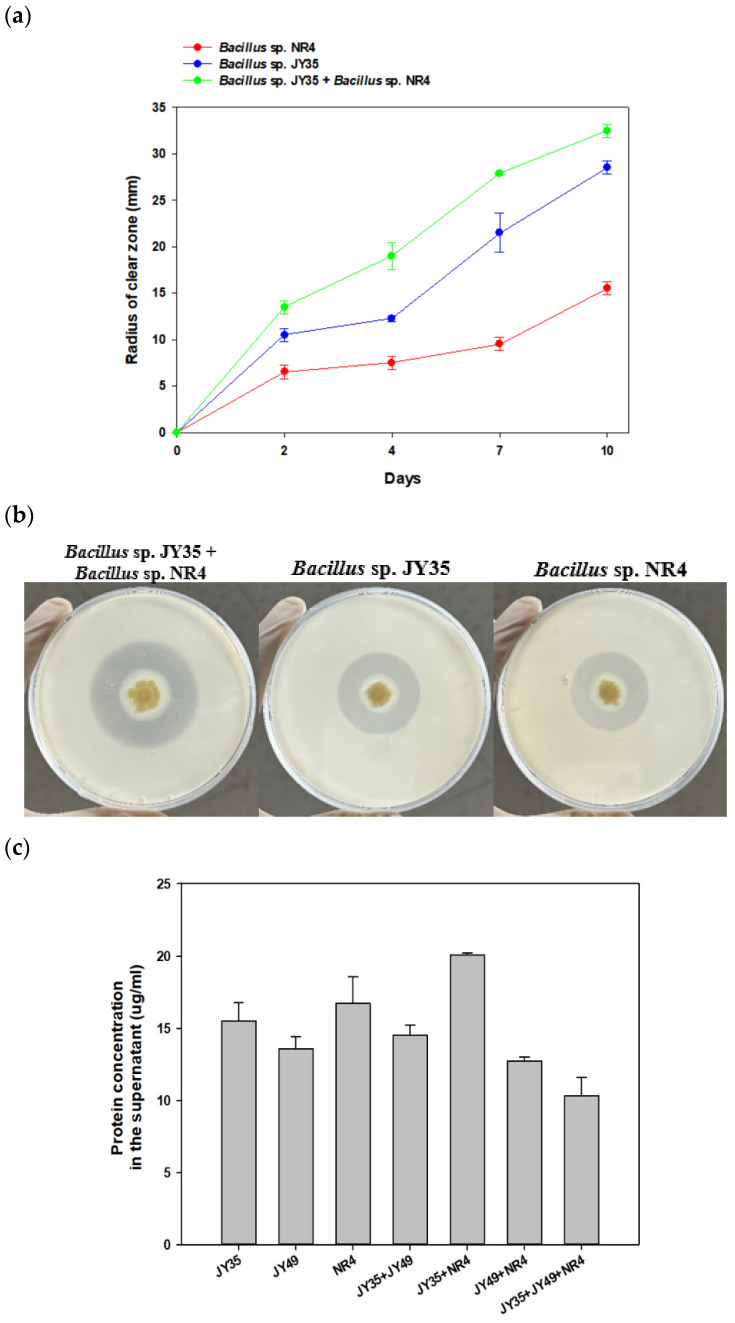
Degradability of consortia, *Bacillus* sp. JY35, and *Bacillus* sp. NR4 in PBS and various bioplastics. (**a**) The radius of the clear zone produced by single strains of *Bacillus* sp. JY35, *Bacillus* sp. NR4, and consortia incubated at 30 °C for 10 days. (**b**) Comparison of clear zone formed by consortia and single strains *Bacillus* sp. JY35 and *Bacillus* sp. NR4 on solid plate containing PBS emulsion. (**c**) Protein concentration (mg/mL) in the supernatant of each strain and consortium.

To confirm the degradation activity of the *Bacillus* sp. JY35 and *Bacillus* sp. NR4 consortium against PBS, a clear zone test was performed on marine broth solid medium containing 1% PBS emulsion for 14 days. From the initiation of clear zone size measurement on day 2, the consortium of *Bacillus* sp. JY35 and *Bacillus* sp. NR4 showed the largest clear-zone radius among the tested strains. This was closely monitored for the subsequent 10 days. In this period, a significant clear zone radius was established in the sequence of the consortium of *Bacillus* sp. JY35 and *Bacillus* sp. NR4. After 10 days of clear-zone testing, the single strain *Bacillus* sp. JY35 had a radius of approximately 28.5 mm, *Bacillus* sp. NR4 showed a radius of approximately 15.5 mm, and the consortium showed a larger radius than each single strain, measuring 32.5 mm (Figure 2a,b). Notably, the consortium exhibited a radius 2.1 times larger than that of *Bacillus* sp. NR4, which formed the smallest clear-zone radius.

To determine the reason for the increased activity of both consortia of *Bacillus* sp. JY35 and *Bacillus* sp. NR4, the protein concentration in each supernatant was quantified using the Bradford assay for the three single strains and four consortia (Figure 2c). Overall, *Bacillus* sp. JY35 and *Bacillus* sp. NR4 exhibited the highest supernatant protein concentration, while the consortium of *Bacillus* sp. JY35, *Terribacillus* sp. JY49, and *Bacillus* sp. NR4 had the lowest. Among the individual strains, the supernatant protein concentration of *Bacillus* sp. NR4 was the highest at 15.60 μg/mL, and the concentration of *Terribacillus* sp. JY49 was the lowest at 14.37 μg/mL. Among the consortia, the supernatant protein concentration of *Bacillus* sp. JY35 and *Bacillus* sp. NR4 was the highest at 20.20 μg/mL, whereas the concentration of the consortium involving the three strains *Bacillus* sp. JY35, *Terribacillus* sp. JY49, and *Bacillus* sp. NR4 was the lowest at 11.77 μg/mL.PBS consists of 1,4-butanediol and succinate monomers. It releases these monomers while degrading PBS, which may affect the growth of the strains. As a result, we confirmed the consumption of monomers and the growth of single strains and consortia through LC analysis and optical density measurement (Figure S1a,b). Consumption for each monomer was compared according to the single strains and consortia. Compared to the control group, consortia of *Bacillus* sp. JY35 and *Bacillus* sp. NR4 and consortia of *Bacillus* sp. JY35, and *Terribacillus* sp. JY49 and single strain *Bacillus* sp. JY35 and *Terribacillus* sp. JY49, all completely consumed succinic acid after 48 h.

### 3.3. Degradation of PBS Film by Consortia over Time and Optimization of Strain Ratio

**Figure 3:**
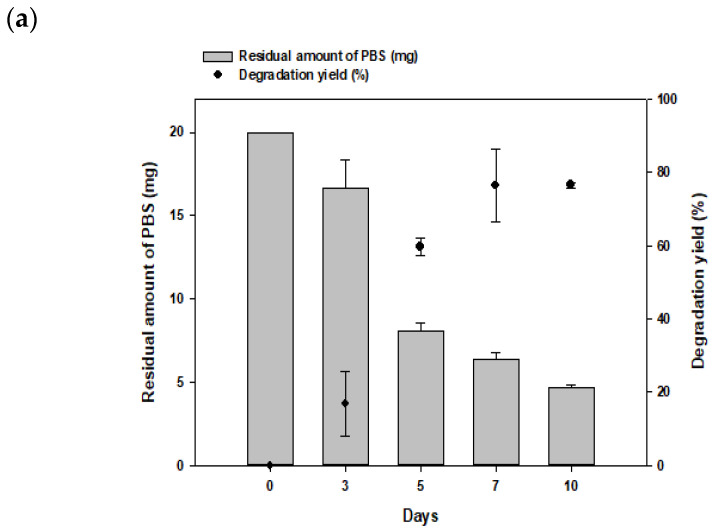

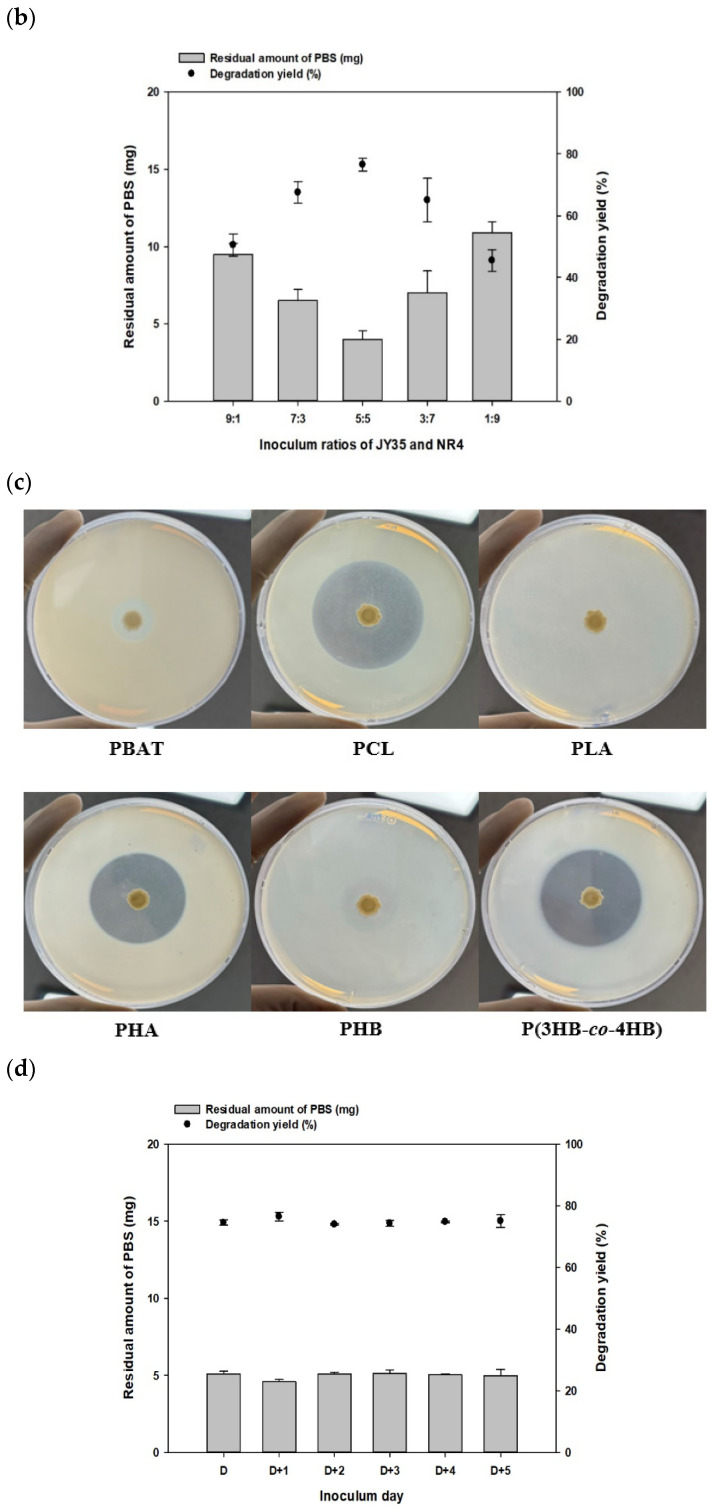
Optimal inoculum ratio for PBS degradation and change in composition by strain of consortia after PBS degradation. (**a**) Comparison of GC–MS results for the PBS degradation yield at different time points of cultivation (0, 3, 5, 7 and 10 days). (**b**) Comparison of GC–MS results for the PBS degradation yield (%) according to inoculum each cell. (**c**) Images of the degradability of consortia for other bioplastics. (**d**) Reproducibility of PBS degradation according to inoculum time point.

To validate the degradation of PBS by the consortium over time, a liquid culture was conducted for 10 days. In this experiment, 20 mg PBS films were added to MB liquid medium, and the experiment was carried out at 30 °C. The degradation yield was approximately 18% on day 3, 58% on day 5, 75% on day 7, and 76.5% on day 10. The degradation yield steadily increased over time, with a significant increase observed from day 3 to day 5 (Figure 3a).

To determine the degradation yield of PBS based on the inoculation ratio of *Bacillus* sp. JY35 and *Bacillus* sp. NR4 in the consortium, the degree of degradation for 10 days for each inoculation ratio (9:1, 7:3, 5:5, 3:7, and 1:9) was confirmed (Figure 3b). For PBS degradation, the inoculation of each cell at a ratio of 5:5 resulted in the highest degradation yield of 76.5%. It was confirmed that the degradation yield decreased as the inoculation ratio of either of the two strains increased or decreased asymmetrically. At ratios of 9:1 and 7:3, the inoculation ratios of *Bacillus* sp. JY35 cells gradually decreased, and the degradation rate of PBS gradually increased with an increase in the ratio of *Bacillus* sp. NR4 cells. The 9:1 ratio showed a degradation yield of 50.5%, the 7:3 ratio showed a degradation yield of 67.5%, the 3:7 ratio showed a degradation yield of 65%, and the 1:9 ratio showed a degradation yield of 45.5%.

The consortium capable of degrading PBS has the potential to degrade other bioplastics such as PBAT, PCL, and P(3HB-*co*-4HB) [22]. We assessed the capacity of the consortium to degrade various bioplastics using clear zone tests. Solid plates containing PBAT, PCL, PLA, PHA, PHB, and P(3HB-*co*-4HB) emulsions were prepared. These plates underwent incubation at 30 °C for 10 days. A combination of *Bacillus* sp. JY35 and *Bacillus* sp. NR4 produced a clear zone on plates containing PBAT, PCL, PHA, PHB, and P(3HB-*co*-4HB) (Figure 3c). In contrast, no clear zones were observed on plates containing PLA. The radius of the clear zone was largest for PCL, followed by P(3HB-*co*-4HB). The clear zone tests facilitated the identification of bioplastics degraded by the consortium of *Bacillus* sp. JY35 and *Bacillus* sp. NR4. Consequently, we confirmed that the consortium that degrades PBS can degrade various bioplastics in addition to PBS.

The repeatability and efficacy of consortia degradation were evaluated under favorable operating conditions by degrading PBS with the consortia at 30 °C. The degradation yield of PBS 7 days after each start date of inoculation was confirmed (Figure 3d). On the first day (D) after inoculation with *Bacillus* sp. JY35 and *Bacillus* sp. NR4, the degradation yield was approximately 74.51% after 7 days. Subsequently, the degradation yield was about 76.5% in the case of new inoculation (D + 1), 74% in the case of new inoculation after 7 days (D + 2), 74.3% in the case of new inoculation after 7 days (D + 3), 74.8% in the case of new inoculation after 7 days (D + 4), and 75.05% in the case of inoculation after 7 days (D + 5). Even when the timing of inoculation of the strains varied, the yield of PBS degradation showed a difference of up to 2.5%, confirming that there was no significant difference. Using the formulated artificial consortia for PBS degradation resulted in efficient PBS degradation with high repeatability and degradation rates.

After 10 days of PBS degradation by a consortium of *Bacillus* sp. JY35 and *Bacillus* sp. NR4, the supernatant of the culture medium was analyzed using GC–MS. The monomer of PBS, 1,4-butanediol monomer, and some related metabolites such as methylpropanoic acid, methylbutanoic acid, and organic acids were detected, suggesting that the consortium of *Bacillus* sp. JY35 and *Bacillus* sp. NR4 degraded PBS to small metabolites [43,44].

### 3.4. Analysis of Chemical and Physical Properties Changes by Consortium

#### 3.4.1. Physical Property Changes of PBS Films Following Degradation by Consortium

As PBS degradation progresses, alterations occur in its physical characteristics [45]. In this study, a degradation experiment on PBS in liquid culture under ideal conditions was performed to assess potential alterations in its physical characteristics. After degradation by a consortium of *Bacillus* sp. JY35 and *Bacillus* sp. NR4, PBS films were examined using SEM and GPC.

**Figure 4:**
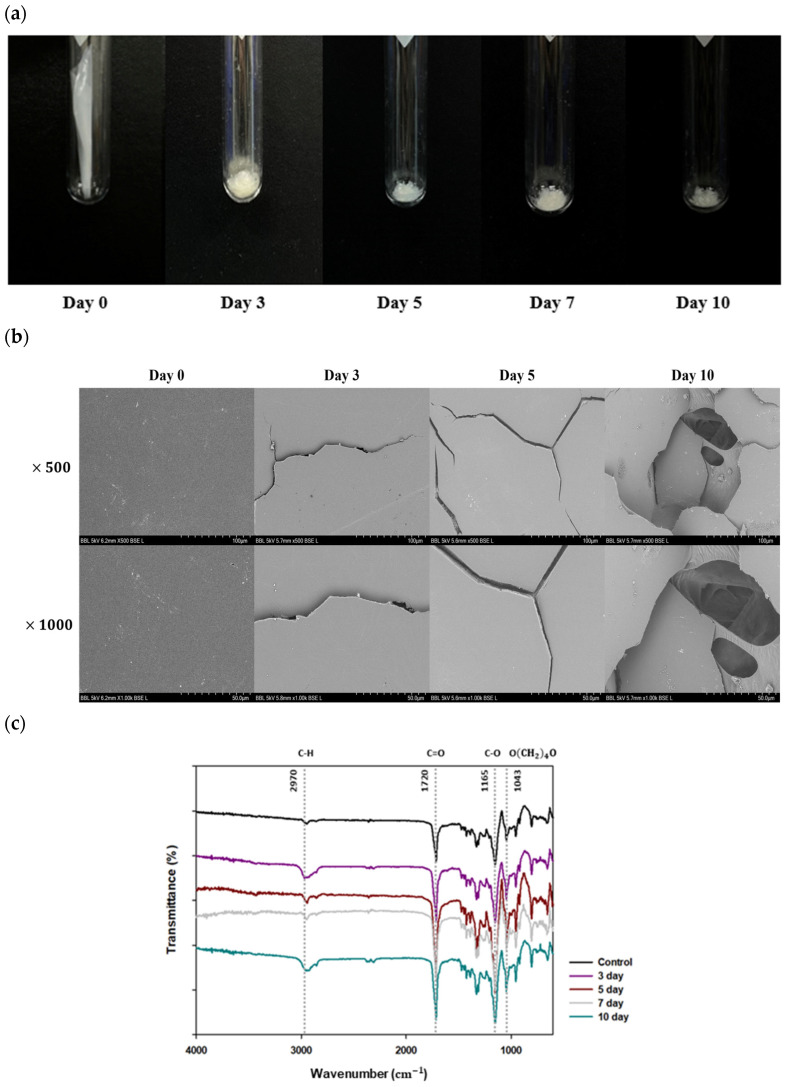
Alterations in the physical and chemical properties of PBS films subsequent to degradation by consortia of *Bacillus* sp. JY35 and *Bacillus* sp. NR4. (**a**) The visual appearance of PBS films undergoes alterations as a result of degradation. Changes in the degradation of PBS films are observed at different incubation periods (0, 3, 5, 7, and 10 days) in MB liquid medium at a temperature of 30 °C. (**b**) Scanning electron microscopy (SEM) images depict the surface changes in PBS films following degradation by consortia of *Bacillus* sp. JY35 and *Bacillus* sp. NR4. The SEM images show the evolving surface morphology of PBS films as degradation progresses over time. (**c**) Functional group analysis using Fourier-transform infrared spectroscopy (FT-IR). FT-IR analysis was used to confirm changes in functional groups. After degradation by consortia, peak intensities changed.

PBS degradation by *Bacillus* sp. JY35 and *Bacillus* sp. NR4 lasted 10 days, and changes in morphology were observed following the lyophilization of the PBS films that remained after degradation on days 0, 3, 5, 7, and 10 (Figure 4a). On day 3 of degradation, it was confirmed that the amount of each PBS film had degraded compared to that before degradation. On day 5, the films were split more than on day 3, and the PBS was considerably degraded. On day 7, continued degradation resulted in finer separated films and a decrease in the amount of each film. On day 10, the amount of film was considerably reduced, and only a small amount remained.

The surface of the PBS film changed as it degraded by the consortium, and SEM was used to observe this surface change (Figure 4b). The PBS film surface was smooth before degradation at the same time. Cracks began to form on the day 3 of degradation. On the day5 following degradation, apparent large cracks appeared on the PBS film. On the day 7, as degradation progressed, many small and fine cracks emerged around one major crack. On the day 10 of degradation, cracks formed further, and a large hole was observed.

Gel permeation chromatography (GPC) was used to verify alterations in the molecular weight ($M_{w}$), of the PBS films because of degradation (Table 3). The weight-average molecular weight ($M_{w}$), number-average molecular weight ($M_{n}$), and polydispersity index (PDI) of the PBS films varied. The $M_{w}$ of PBS films decreased from 9.49 × 10^4^ to 4.06 × 10^4^ during degradation, while the $M_{n}$ also decreased from 3.87 × 10^4^ to 2.05 × 10^4^. The PDI serves as an indicator of the breadth of the molecular weight distribution. A higher PDI value indicates a wider range of molecular weights [46]. A decrease in the PDI suggests a narrowing of the molecular weight range. Microbial degradation can occur as high-molecular-weight chains are degraded into smaller and more consistent fragments [47]. The confined PDI values remained relatively constant as the PBS degraded, suggesting that degradation occurred uniformly across the surface of the PBS film.

#### 3.4.2. Chemical Property Changes of PBS Films following Degradation by Consortium

Fourier-transform infrared spectroscopy was employed to investigate alterations in the functional groups of the PBS films following degradation by the consortium (Figure 4c). The FT-IR spectra indicated differences in peak intensities, indicating that functional groups were modified during the degradation process by *Bacillus* sp. JY35 and *Bacillus* sp. NR4. The consortia were cultivated with PBS films for 10 days, and the leftover PBS films were collected. Following PBS degradation, a noticeable change was observed in the peak intensity at 2970 cm^−1^, corresponding to the C–H stretching bond. This alteration suggested a variation in the alkane groups. Additionally, changes in the peak intensities at 1720 cm^−1^ and 1165 cm^−1^, corresponding to C=O stretching and C–O ester bonds, respectively, were confirmed [48,49]. These bonds are representative of ester bonds present in PBS. The alterations in these two peaks signify the degradation of PBS by the consortium. Furthermore, the peak at 1043 cm^−1^ indicated the O(CH_2_)_4_O vibration, a functional group inherent in PBS structures. Therefore, the degradation induced by the consortium of *Bacillus* sp. JY35 and *Bacillus* sp. NR4 results in a significant change in the chemical structure of PBS.

## 4. Conclusions

Using consortia is crucial because degrading PBS with a single strain is challenging. Therefore, identifying strains that positively influence each other during degradation and forming appropriately formulated artificial consortia are essential. This study aimed to enhance PBS degradation by constructing a consortium of three strains: *Bacillus* sp. JY35, which degrades PBAT; *Terribacillus* sp. JY49, a PBS-degrading strain; and *Bacillus* sp. NR4, a PCL-degrading strain. To enhance the consortia reproducibility and degradation reproducibility by using a small number of strains, two strains, *Bacillus* sp. JY35 and *Bacillus* sp. NR4, were used to construct consortia, and degradation tests were performed. Although *Bacillus* sp. JY35 and *Bacillus* sp. NR4 exhibited low degradability in PBS individually, the consortium of two strains significantly increased the yield of PBS degradation. The co-culture system of these bacteria appeared to promote plastic degradation and the consortium of *Bacillus* sp. JY35 and *Bacillus* sp. NR4 exhibited the highest PBS degradation yield (76.5%) for 10 days. GC–MS, SEM, GPC, and FT-IR analyses confirmed the PBS degradation ability of the *Bacillus* sp. JY35 and *Bacillus* sp. NR4 consortium. This study highlights the efficient degradation of PBS by the *Bacillus* sp. JY35 and *Bacillus* sp. NR4 consortia, which are crucial for maintaining degradation activity through a consortium comprising a small number of strains, such as these two. This approach ensures consortia with reproducible degradation rates. Furthermore, it is proposed that strains with low degradation activity can contribute to plastic degradation when organized into consortia. This suggests that even bacteria with individually low plastic degradation capabilities can play a significant role in the overall efficiency of plastic degradation when working collaboratively in consortia.

## Figures and Tables

**Table 1:** Previous reports of PBS degradation by a single strains and consortia of bacteria.

Strain	Temperature	Days of Degradation	Degradation Percentage	Ref.
*Fusarium solani*	-	14	2.8 wt%	[20]
*Laceyella sacchari LP175*	50 °C	2	46.5 wt%	[21]
*Terribacillus goriensis*	30 °C	10	31.4%	[22]
*Bionectria ochroleuca BFM-X1*	30 °C	30	60 wt%	[23]
*Aspergillus versicolor*, *Penicillium*, *Bacillus*, *Thermopolyarpora*	-	90	60.7%	[25]
*Bacillus* sp. JY35, *Bacillus* sp. NR4	30 °C	10	76.5%	In this study

**Table 2:** Strains used in this study.

Strains	Target	Degradability for PBS *	Temperature	Day of Degradation	Degradation Yield (%)	Ref
*Bacillus* sp. JY35	PBAT	+	30 °C	21 days	50%	[31]
*Terribacillus* sp. JY49	PBS	++	30 °C	10 days	22.3%	[22]
*Bacillus* sp. NR4	PCL/PBAT	+	37 °C	14 days	88.3% (PCL 85%, PBAT 15%)	[32]

* +: PBS degradation yield (%) of not more than 30%, ++: PBS degradation yield (%) greater than 30%.

**Table 3:** Molecular weight of PBS films, analyzed using gel permeation chromatography.

Day	$M_{n}$ × $10^{4}$	$M_{w}$ × $10^{4}$	$M_{w}$$/M_{n}$ (PDI)
0	3.87	9.49	2.45
3	2.19	4.60	2.10
5	2.10	4.53	2.15
7	2.08	4.28	2.06
10	2.01	4.06	2.05

## Data Availability

Data are contained within the article and Supplementary Materials.

## Supplementary Materials

The following are available online at https://www.mdpi.com/article/10.3390/polym16050651/s1, Figure S1: Comparison of succinic acid and 1,4-butanediol degradation by single strains and consortium (a) Residual concentration after consumption for 48h of succinic acid and 1,4-butanediol were tested via LC analysis. (b) Comparison of consortia growth after adding two monomers.
