# Supplementary material for: Reproducible Polybutylene Succinate (PBS)-Degrading Artificial Consortia by Introducing the Least Type of PBS-Degrading Strains
Source: Polymers (Basel). 2024 Feb 28;16(5):651. doi: 10.3390/polym16050651 (PMC10934136; doi:10.3390/polym16050651)
Supplement: Supplementary file 1 [file polymers-16-00651-s001.zip › polymers-2856985-supplementary.pdf]

Supplementary Material

Reproducible Polybutylene Succinate (PBS)-Degrading Artificial Consortia by Introducing the Least Type of PBS-Degrading Strains

Nara Shin [1], Su Hyun Kim [1], Jinok Oh [1], Suwon Kim [1], Yeda Lee [1], Yuni Shin [1], Suhye Choi [1], Shashi Kant Bhatia [1,2], Yun-Gon Kim [3] and Yung-Hun Yang [1,2,*]

[1] Department of Biological Engineering, College of Engineering, Konkuk University, Seoul 05029, Republic of Korea; dksk71@naver.com (N.S.); gsm06136@naver.com (S.H.K.); xmfvm@naver.com (J.O.); rlatn990@naver.com (S.K.); karecurry@konkuk.ac.kr (Y.L.); sdbsdl0526@naver.com (Y.S.); suhye0823@konkuk.ac.kr (S.C.); shashikonkukuni@konkuk.ac.kr (S.K.B.)

[2] Institute for Ubiquitous Information Technology and Application, Konkuk University, Seoul 05029, Republic of Korea

[3] Department of Chemical Engineering, Soongsil University, Seoul 06978, Republic of Korea; ygkim@ssu.ac.kr

* Correspondence: seokor@konkuk.ac.kr; Tel.: +82-10-9079-9846

(a)

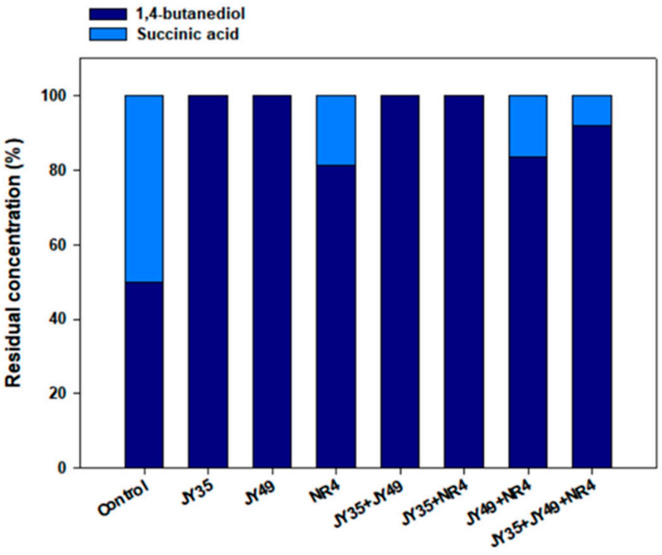

(b)

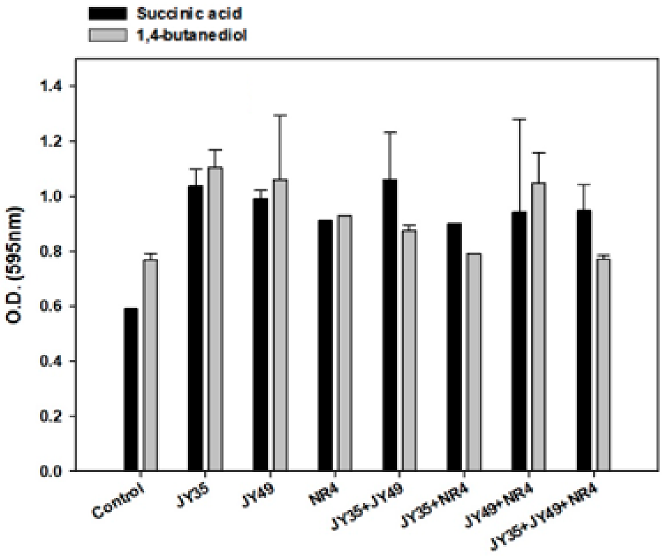

Figure S1. Comparison of succinic acid and 1,4-butanediol degradation by single strains and consortium (a) Residual concentration after consumption for 48h of succinic acid and 1,4-butanediol were tested via LC analysis. (b) Comparison of consortia growth after adding two monomers.
